# Impact of Climate Change on the Global Dynamics of Vector-Borne Infectious Diseases: A Narrative Review

**DOI:** 10.7759/cureus.77972

**Published:** 2025-01-25

**Authors:** Esteban Zavaleta-Monestel, Carolina Rojas-Chinchilla, Paula Molina-Sojo, Maria Fernanda Murillo-Castro, Jose Pablo Rojas-Molina, Ernesto Martínez-Vargas

**Affiliations:** 1 Research, Hospital Clínica Bíblica, San José, CRI; 2 Pharmacy, Hospital Clínica Bíblica, San José, CRI; 3 Pharmacy, Universidad de Iberoamérica, San José, CRI

**Keywords:** dengue, lyme's disease, malaria, s: climate change, vector-borne diseases, zika infection

## Abstract

Climate change has significantly altered the dynamics of vector-borne infectious diseases, favoring their proliferation and geographic expansion. Factors such as rising temperatures, the frequency of extreme weather events, and uncontrolled urbanization have increased the incidence of diseases such as dengue, Zika, chikungunya, malaria, and Lyme disease, especially in vulnerable regions with limited infrastructure. This article presents a narrative review based on recent scientific literature (2018-2025) to assess the impact of climate change on vector distribution, co-infections, and control strategies. The evidence collected highlights how changing climate conditions, combined with socioeconomic, political, and demographic factors, exacerbate public health crises and complicate mitigation efforts. It is concluded that facing this challenge requires a comprehensive strategy that combines environmental management, technological innovation, epidemiological surveillance, and community educational programs, promoting a coordinated global response to reduce the associated risks.

## Introduction and background

Climate change is currently one of the most pressing threats to human health and to almost all biological species on the planet. Constant global warming, driven largely by excessive anthropogenic greenhouse gas emissions, has the potential to alter aspects of daily life, increasing the incidence of disease as the life cycles of pathogens such as dengue virus, Zika, and chikungunya become more sensitive to environmental factors such as soil, air, and water [1,2].

This phenomenon also influences the proliferation of disease vectors, such as mosquitoes, ticks, and sandflies, which thrive in warmer, wetter conditions. In the case of *Aedes aegypti*, studies have shown that climate change is expected to increase the global land area suitable for proliferation. Specifically, it is expected that the climatically suitable area for *Aedes aegypti *increase by 8% to 13% by 2061-2080 compared to current conditions [3]. For example, *Aedes aegypti* and *Aedes albopictus*, the main vectors of diseases such as dengue, chikungunya, and Zika, have expanded to regions previously unsuitable for survival, including parts of Europe and North America [4]. This expansion is associated with an increase in the number of people exposed, with projections indicating an increase of 298-460 million people (8%-12%) due to climate change alone [3].

In addition, rising global temperatures and the increased frequency of extreme weather events, such as floods, droughts, and heat waves, have favored the spread of diseases such as malaria and Lyme disease to new regions, including high latitudes that were initially considered inhospitable [5-7]. This reality is exemplified by recent data from South Asia, where malaria cases increased fivefold following severe flooding in 2022, highlighting the vulnerability of these regions to climate-induced health crises [8].

In the last decade, the prevalence of diseases such as dengue, Zika, chikungunya, malaria, and Lyme disease has increased considerably. This increase has been observed especially in areas of sub-Saharan Africa, South Asia, and parts of Latin America, where public health resources and infrastructure are insufficient to address these threats. If sufficient action is not taken, this trend is likely to worsen. For example, projections suggest that Lyme disease cases in Canada could range from 120,000 to 500,000 annually by 2050 due to the expanding range of *Ixodes scapularis* ticks [9]. Similarly, studies conducted in South Korea have documented an increase in tick-borne infections, demonstrating how climate change influences their proliferation even under stable socioeconomic conditions, due to factors such as increased temperature and humidity [10].

As a result of the increase in vector prevalence, transmission seasons are expected to be longer and the geographical distribution of diseases such as dengue, Zika, and chikungunya is expected to expand. For example, the optimal temperature range for the transmission of these diseases is between 18°C and 34°C, with efficacy peaks between 26°C and 29°C. These conditions, increasingly prevalent in temperate regions, also imply increased risks of simultaneous transmissions of multiple pathogens [11].

The economic burden of vector-borne diseases is also significant, costing approximately $12 billion annually. Developing countries, where public health systems are often underfunded, and where there is insufficient economic development to cope with them, are particularly vulnerable. Socioeconomic factors, such as poverty, limited access to health care, and education of the population, aggravate the impact of these diseases in these regions [12].

In this context, advances in scientific research and predictive modeling have played a crucial role in improving our understanding of the relationship between climate change and the spread of disease. For example, climate variables combined with machine learning techniques have been used to predict dengue risk in Costa Rica, illustrating the potential of these tools to guide targeted interventions. However, a significant gap persists in global epidemiological surveillance, particularly in low-income regions disproportionately affected by climate change [7,13].

This narrative review aims to comprehensively assess the impact of climate change on the dynamics of vector-borne infectious diseases. It explores migration patterns and changes in disease prevalence and identifies control and mitigation measures implemented globally. By integrating recent global statistics and evidence from diverse geographic regions, this work seeks to provide a solid understanding of how climate change reshapes global health landscapes.

## Review

Methodology

This article is a narrative review focused on assessing the impact of climate change on vector-borne diseases, considering vector distribution, disease prevalence, and challenges related to co-infections. The process of preparing this review followed a structured methodology to ensure the completeness and reproducibility of the results.

Search strategy

A systematic search was conducted in recognized electronic databases, including PubMed and Google Scholar, using the following search terms: "climate change," "vectors," "dengue," "zika," "chikungunya," "lyme disease," and "malaria." Boolean operators (AND, OR, NOT) were used to combine terms and refine the search. Filters were applied to select publications in English and Spanish, published between 2018 and 2025, with an emphasis on recent and relevant studies.

Inclusion and exclusion criteria

We included studies assessing the impact of climate change on vector proliferation, the increase in infectious diseases, and control or mitigation strategies. We also considered systematic reviews and epidemiological studies that provided data from various geographic regions and excluded non-peer-reviewed reports, duplicate articles, studies focused on specific diseases not directly related to climate change, and publications outside the established time range.

Method of analysis

Data extracted from the selected studies were categorized according to their thematic relevance, dividing them into sections that included vector distribution, co-infections, and control methods. The references were checked to ensure their validity and timeliness, covering a spectrum of regional and global perspectives.

Selection process

**Figure 1:**
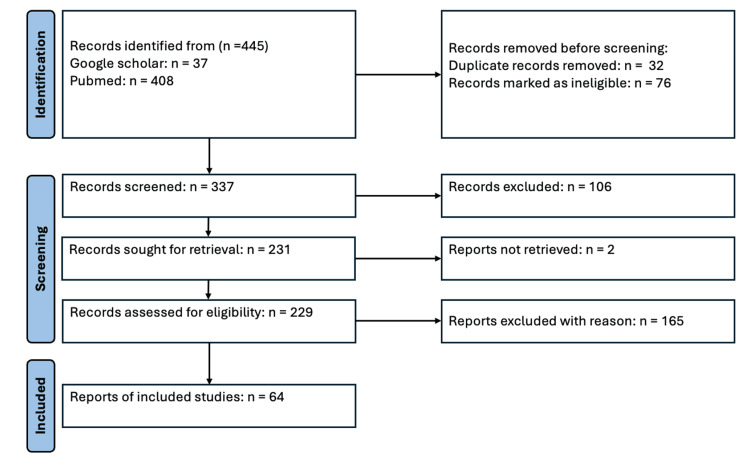
Flowchart of search results and selection of relevant articles

Initially, 445 publications were identified. After removing duplicates and performing an analysis of titles and abstracts, 229 articles were selected for full review. Finally, 64 studies met the inclusion criteria and formed the basis for narrative synthesis. A flowchart details the selection and screening process (Figure 1).

Impact of climate change on vectors and diseases

Climate change is profoundly transforming the geographic distribution of vector-borne diseases, exacerbating their impact on global public health. Factors such as rising temperatures, high humidity, and extreme weather events favor the proliferation of vectors such as mosquitoes and ticks, increasing the risk of outbreaks in previously unaffected regions. Specific climate variables such as average temperature, relative humidity index, cumulative rainfall, frequency of heavy rainfall, and length of wet seasons have been identified as key predictors for the proliferation of diseases such as dengue [14,15].

In sub-Saharan Africa, climate change significantly affects diseases such as dengue as it expands the geographic range of vectors such as dengue fever, *Aedes aegypti*, especially in West and Central Africa. This increase in geographic distribution also affects other species, such as *Aedes vittatus* and *Aedes luteocephalus*, which thrive in warmer and wetter climate conditions, exacerbating challenges in countries such as Cameroon and the northern Democratic Republic of Congo [16]. On the other hand, in Southeast Asia, densities are expected to increase *Aedes aegypti *and *Aedes albopictus *significantly, with projections of up to a 46% increase by the end of the century in highly vulnerable areas. This increase not only increases dengue transmission but also the risk of co-infection with other pathogens, complicating control efforts in countries such as Thailand, Indonesia, and the Philippines [17].

The coexistence of multiple vector-borne diseases, such as dengue, Zika, and chikungunya, poses unique challenges for diagnosis and treatment. Since these diseases share similar initial symptoms, such as fever, headache, and rashes, healthcare professionals face difficulties differentiating them, which can delay proper treatment. This complexity is exacerbated in areas with limited resources, where diagnostic tools are scarce or non-existent. Co-infections also create therapeutic uncertainties, as treatments for one disease may not be equally effective or even harmful to others [14,18].

For example, during arbovirus outbreaks in Latin America and Southeast Asia, cases of simultaneous transmission of dengue and Zika have been documented, increasing the burden on health systems. This highlights the need to strengthen diagnostic capacity through specific tests, such as polymerase chain reaction (PCR) and enzyme-linked immunosorbent assay (ELISA) methods, as well as the implementation of more robust epidemiological surveillance systems. In sub-Saharan Africa, improved entomological surveillance, along with educational campaigns on preventive measures, could mitigate the impact of these diseases [19-21].

Malaria cases are projected to increase in regions previously too cold to host transmitting vectors, such as the northern United States, Scandinavia, and northern Europe. These areas, where malaria outbreaks were atypical, now, with the increases in global temperature experienced in recent decades, present favorable conditions due to the increase in temperatures and rainfall [22].

In regions such as Costa Rica, elimination is close to being achieved, thanks to effective health policies that include the introduction of supervised treatments with chloroquine and primaquine since 2006. This has reduced annual malaria cases by 98% between 2009 and 2018 [23]. However, malaria transmission remains sensitive to extreme weather events and natural disasters, underscoring the importance of timely diagnosis and treatment, as well as improving living conditions in affected areas [23].

Similarly, Lyme disease has shown a significant increase in Canada, driven by the expansion of the tick's range *Ixodes scapularis*. Warmer temperatures have prolonged their active season and favored their survival in more northern latitudes. This change has increased the risk to human populations in these areas, where cases were previously sporadic [24].

These examples underscore the importance of comprehensive strategies that combine environmental management, entomological surveillance, and access to timely diagnostics to address the challenges posed by climate change in the global dynamics of vector-borne diseases [25].

Change in vector distribution

Climate change is profoundly transforming the geographical distribution of vectors responsible for disease transmission, generating variations that depend on both local climatic conditions and socioeconomic factors. Rising temperatures, changes in precipitation patterns, and the increased frequency of extreme weather events have favored the expansion of vectors such as *Aedes aegypti*, *Aedes albopictus*, *Ixodes scapularis*, and *Ixodes ricinus*, among others. These alterations have increased the risks of diseases such as dengue, chikungunya, Zika, malaria, and Lyme disease in various regions of the world, where many of these problems were previously unknown or controlled [26,27].

In tropical and subtropical regions of Latin America, Africa, and Asia, hot and humid conditions favor the proliferation of *Aedes aegypti*, which has led to recurrent outbreaks of diseases such as dengue, chikungunya, and Zika. However, climate change is intensifying this problem by increasing the average temperature, relative humidity, and length of rainy seasons, which expands the geographic distribution of these diseases [28,29]. It is estimated that by the end of the twelfth century, there will be an increase of 8% to 13% in the land area affected by this vector. These conditions also accelerate the reproductive cycles of vectors and prolong transmission periods, increasing the incidence of infections, especially in urban and rural areas of Central and South America [30-32].

**Figure 2:**
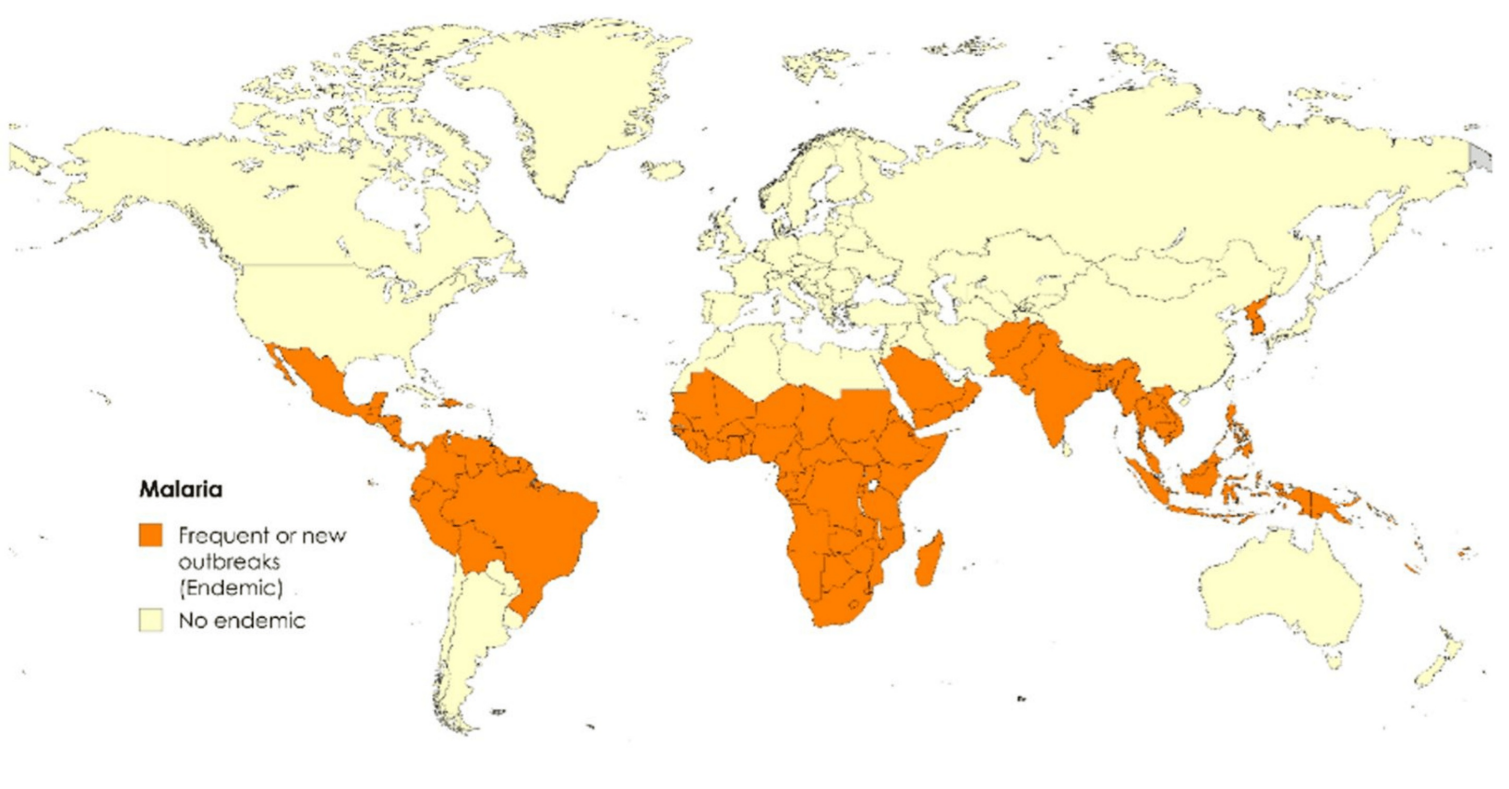
Geographical distribution of malaria: endemic and non-endemic regions, with a presence in the latter attributed to modifications in vector habitats Image created by the authors

Malaria represents a significant burden on health systems in Africa, especially in sub-Saharan African countries (Figure 2), where changes in average temperatures and rainfall have contributed to an increase in its incidence [33]. Temperatures between 20°C and 30°C are ideal for the Anopheles mosquito development, so an increase in average temperatures expands breeding habitats and prolongs transmission seasons [32,34].

Extreme weather events, such as flooding, have reactivated previously controlled outbreaks and transformed inhospitable areas into vector-friendly environments, allowing malaria to spread to previously unaffected areas, such as the highlands of Ethiopia and Kenya, as well as regions of North America and Europe, where cases were previously rare or non-existent [35,36].

An illustrative case is that of Costa Rica, a country with a solid health system that was close to eradicating malaria, with an eradication of 99%, with only the border areas with Panama and Nicaragua being affected. However, in recent years, the increase in rainfall and the frequency of floods has favored the reappearance of the disease, increasing cases even in regions where effective control had previously been achieved and no infections were recorded, a phenomenon that underscores the vulnerability of previously controlled areas to the effects of climate change [14].

The situation in South and Southeast Asia presents a unique challenge due to accelerated population growth and migration to large cities, which increases population density and creates conditions conducive to vector proliferation and disease transmission. This phenomenon, common in countries with high population growth rates such as India, is exacerbated by uncontrolled urban sprawl and lack of proper planning, which often leads to poor drainage and sanitation systems. These infrastructural deficiencies favor the creation of vector breeding sites, intensifying public health crises in a region already vulnerable to vector-borne diseases [37-39]. In addition, political instability in these areas leads to population displacements, often resulting in precarious residential settlements with inadequate infrastructure and sanitation conditions. These circumstances favor the proliferation of vectors and the transmission of diseases while further hindering the ability of health systems to respond effectively [40].

Poverty and socioeconomic factors play a key role in vector distribution. In resource-limited regions, such as sub-Saharan Africa, and certain regions of Asia and Central America, the lack of adequate infrastructure, including drainage and sanitation systems, and restrictions on access to health services exacerbate the problem [41]. These limitations hinder surveillance, diagnosis, treatment, and implementation of essential preventive measures, such as the use of insecticide-impregnated bed nets, key to effective control, as well as the use of tools such as remote sensing and geographic information systems. However, educating the population is presented as a viable and transformative solution in the fight against vector-borne diseases, especially in regions with limited resources. Informing communities about simple preventive practices, such as eliminating mosquito breeding sites, properly managing standing water, and using personal protective measures such as mosquito nets and repellents, can significantly reduce vector proliferation. In addition, educational programs not only strengthen awareness of the impact of climate change on health but also encourage the active participation of communities in prevention, decreasing the burden of disease and building resilience in the face of future health challenges [42,43].

In North America and Europe, global warming has allowed the establishment of vectors in areas previously inhospitable to their survival, such as regions of extreme altitudes and latitudes near the poles. Although these areas are mostly highly economically developed, accelerated urbanization, combined with the increasingly intense effects of climate change, has increased population density, replicating a situation similar to that of regions with fewer resources. This increase in the susceptible population, together with higher temperatures and higher relative humidity, has created ideal conditions for the reproduction of transmitting vectors, favoring the spread of diseases in these areas [11].

**Figure 3:**
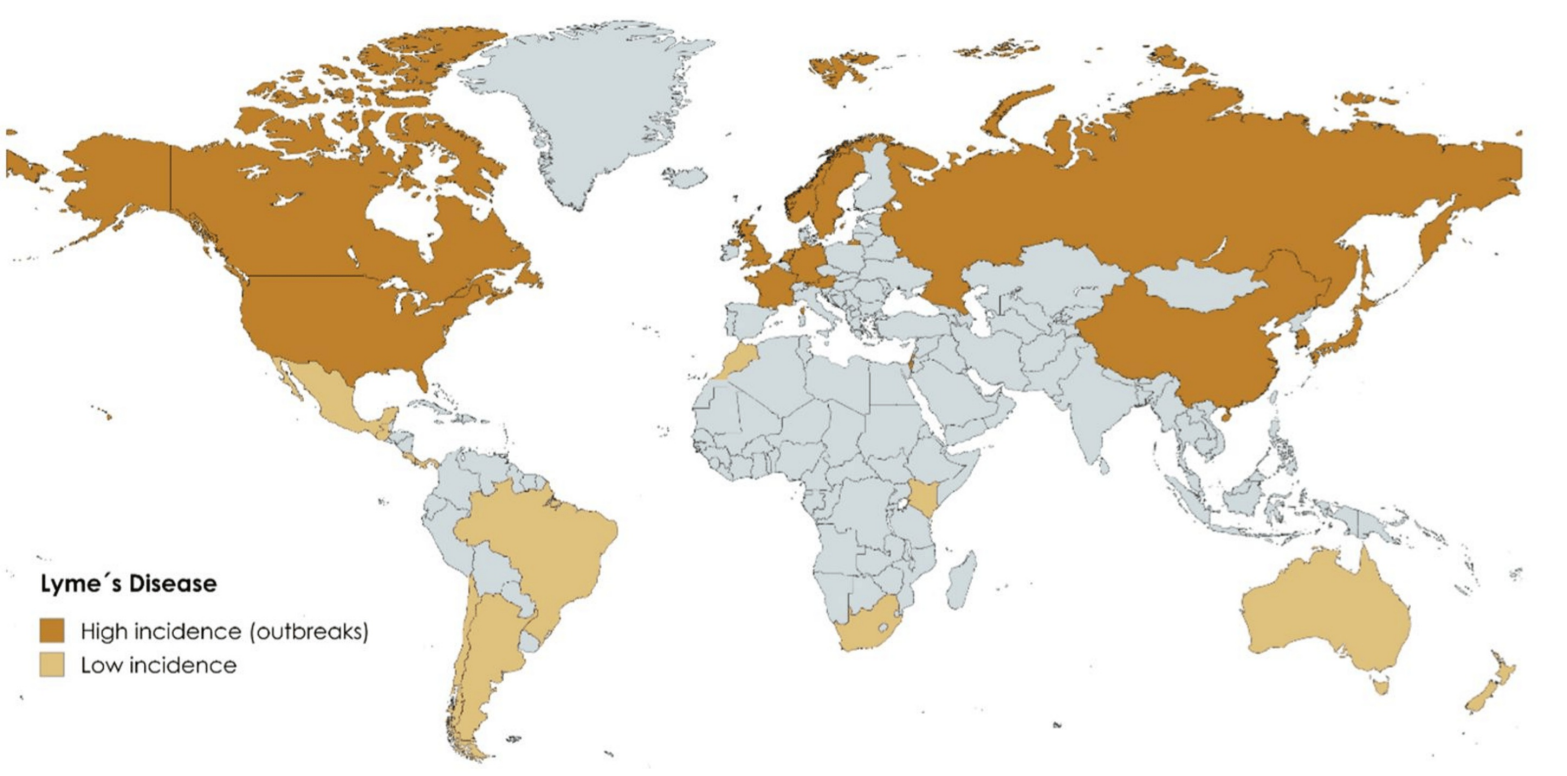
Geographical distribution of the areas most affected by the tick that transmits Lyme disease Image created by the authors

*Aedes aegypti *and *Aedes albopictus* have expanded their habitat into temperate zones, mainly in the states of Texas and Florida, where less severe winters have allowed the proliferation of native mosquitoes, increasing the risk of transmission [44]. However,* Ixodes scapularis*, a vector of Lyme disease, has increased its presence in the northern United States and mainly Canada, as well as in northern regions of Europe (Figure 3) [45].

Lyme disease in Canada is significantly affected by the tick's northward spread *Ixodes scapularis*, especially in densely populated areas such as Ottawa and Ontario [45]. In these regions, the establishment of the populations of this vector has been documented, whose distribution is expanding at an estimated rate of 18 km per year, favored by increasingly suitable climatic conditions. This phenomenon could trigger a considerable increase in the incidence of Lyme disease, with projections estimating between 120,000 and more than 500,000 cases annually by 2050 in Canada, which would represent a significant economic impact [46].

The Canadian government has implemented rigorous measures to address Lyme disease, including thorough monitoring of cases to identify areas of risk and track the tick's geographic expansion *Ixodes scapularis*. At the same time, educational campaigns are carried out aimed at both the general public and health professionals, with the aim of increasing awareness about the prevention, early diagnosis, and appropriate treatment of this disease [45].

In response to vector-related challenges, integrated strategies for vector control are being developed. In Europe and North America, the use of sterilized male mosquitoes has shown efficacy in reducing mosquito populations (*Aedes albopictus*). However, these initiatives face limitations, such as high costs and the need for long periods of time to achieve meaningful results [47].

Problems with co-circulating diseases

In regions affected by climate change, variations in temperature, humidity, and precipitation have facilitated the expansion of vector habitats into previously non-endemic areas. This phenomenon has significantly increased human exposure to multiple pathogens simultaneously, increasing the likelihood of co-infections. This is particularly evident in tropical regions, where the same host can be infected by cocirculating pathogens, such as dengue, chikungunya, and Zika arboviruses, all of which are transmitted by mosquitoes* Aedes aegypti*. Studies have shown that this vector can carry and transmit several arboviruses simultaneously, complicating both the diagnosis and treatment of infections [48,49].

The interaction between these viruses is complex. In some cases, one pathogen can interfere with the replication of another or activate innate immunity responses that modulate the infection. However, adverse effects can also be generated, such as immunosuppression caused by one pathogen that facilitates the replication of another, which enhances the severity of the disease. In addition, the overlapping of symptoms common to various vector diseases, such as fever, headache, and myalgia, makes it difficult to accurately identify the agents involved [48,49].

A relevant example is the co-infection of malaria and dengue. In these cases, *Plasmodium *(a parasite that causes malaria) alters immune function due to the destruction of red blood cells and systemic inflammation, while the dengue virus causes thrombocytopenia and dysregulation of the inflammatory response. In co-infected patients, immunosuppression caused by malaria may facilitate more aggressive viral replication of dengue, while dengue-associated thrombocytopenia aggravates the bleeding complications of malaria. This scenario increases the clinical severity and complicates treatment, since antimalarial drugs such as quinine, artemisinin, chloroquine, and hydroxychloroquine can exacerbate thrombocytopenia, increasing the risk of serious complications [50,51].

On the other hand, co-infections between Zika and dengue represent a paradigmatic case of immunological interactions. These infections can trigger a cross-response, increasing the severity of the disease through the phenomenon of antibody-dependent potentiation (ADE). This mechanism not only intensifies the complications of dengue but also increases the risk of Zika-associated fetal harm in pregnant women. In addition, serological tests, such as ELISA and those based on IgM/IgG, are often cross-reactive, generating false-positive results that make diagnosis and clinical management difficult, especially in vulnerable patients such as pregnant women [52,53].

Mitigation and control methods

Strategies to mitigate vector-borne diseases encompass a wide range of interventions designed to reduce the transmission of these diseases. These strategies can be grouped into various categories, with environmental management being a key element. In this context, climate change control and related actions play a fundamental role in reducing habitats conducive to vectors. In addition, individual measures are included, which include preventive practices that each person can adopt to reduce the risk of contagion and spread of the disease. Finally, chemical control and integrated vector management stand out, which combine different approaches to maximize the effectiveness of vector control.

Environmental management

Climate change has proven to be a determining factor in the expansion and proliferation of vector-borne diseases, so effective environmental action is essential. Reducing greenhouse gas emissions through renewable energy, energy efficiency, and reforestation is critical to mitigating their impact. Water and sanitation management, such as improving infrastructure to avoid stagnation and ensure safe drinking water, prevent breeding sites for vectors such as *Aedes aegypti *[54].

Sustainable urban planning and conservation of natural ecosystems limit human-vector interaction, while protected reserves and sustainable agricultural practices contribute to environmental stability. Resilient infrastructure, such as adapted drainage systems, strengthens the capacity to respond to extreme weather events [55].

Personal measures and education of the population

Control measures include individual actions that contribute significantly to reducing the transmission of vector-borne diseases. The use of insecticide-impregnated mosquito nets and treated materials, such as curtains, has proven to be highly effective in decreasing the density of vectors responsible for diseases such as malaria and dengue. These tools are critical in vector control programs, as they effectively interrupt the transmission cycle [56].

In addition, insecticide-treated clothing and the application of topical repellents are widely used complementary personal protection measures, especially in areas of high endemicity. These strategies form an effective barrier against vector bites and, when implemented together, enhance the prevention and control of these diseases [55].

On the other hand, educational interventions have proven to be key to increasing knowledge and encouraging protective behaviors in communities. A systematic review showed that educational programs generated significant improvements in understanding of the problem and self-reported protection practices. These initiatives often focus on eliminating mosquito breeding sites, an essential component in prevention. By educating communities about the importance of keeping areas free of standing water, covering containers, and properly managing solid waste, personal protection measures such as the use of mosquito nets and repellent strengthen the collective response capacity to reduce the presence of vectors and associated diseases [57].

Chemical control

Insecticide application, whether through residual sprays indoors or outdoors, continues to be a critical component in vector control strategies. However, the effectiveness of these interventions is intrinsically linked to their correct implementation and coverage. A critical challenge associated with this measure is the development of insecticide resistance by vectors, underscoring the need to investigate and develop alternative strategies that complement and enhance existing interventions [58,59].

Biological and genetic control

There are several genetic and biological control strategies that have been tried to be put into practice that seek to reduce the impact that vectors and vector-borne diseases can produce. Recently, symbiotic species such as bacteria of the genus *Wolbachia *have been used [60]. These bacteria infect a wide variety of arthropods, including insects and some nematodes. These bacteria have been introduced as a strategy to reduce the vectorial capacity of mosquitoes Aedes aegypti, since these bacteria compete with viruses or parasites within the mosquito's body, limiting its replication [60,61].

Advanced gene drive technologies have also been developed and applied to propagate genetic modifications in mosquito populations with the aim of suppressing or replacing them. These technologies take advantage of molecular tools to introduce genetic changes that affect the viability or fertility of vectors, which reduces their ability to reproduce and, consequently, population density [61].

In addition, techniques for the release of sterilized males have been implemented using chemical, radiological, or genetic methods. These males, when mating with females in the wild, generate non-viable offspring, progressively decreasing mosquito populations. Both strategies have significant advantages by focusing exclusively on vectors, minimizing adverse impacts on the ecosystem [62].

Technological innovation

Nanotechnology offers innovative tools for vector control, providing more specific and sustainable methods than traditional ones. Its applications include nanoparticles for the controlled release of insecticides, which increases efficiency and reduces environmental impact, and nanosensors for the early detection of outbreaks. However, the implementation of these technologies in low-resource settings presents significant challenges. Limited access to advanced infrastructure, high upfront costs, and lack of technical expertise can make it difficult to adopt it widely. In addition, ensuring the safe and ethical deployment of these technologies requires addressing regulatory gaps and building trust in communities [63].

In this context, community participation and education play a fundamental role in overcoming these barriers. Informing and engaging local populations about the benefits and potential risks of nanotechnology can promote their acceptance and collaboration.

## Conclusions

Climate change has significantly transformed the dynamics of vector-borne diseases, expanding their geographical distribution, increasing their prevalence in previously less affected areas of the world, and prolonging transmission seasons. This phenomenon of displacement of vector habitats to new regions due to changes in climatic conditions has increased the risk of co-infections, which poses additional challenges in diagnosis and treatment, complicating the response capacity of health systems. In this context, the implementation of comprehensive strategies such as environmental management, the development of innovative technologies, and the improvement of epidemiological surveillance systems is essential to mitigate the impact of these diseases. It is also key to promote community education and individual preventive interventions to reduce the associated risks. Overcoming these challenges requires coordinated action at the global level that includes both adaptation to new climatic conditions and mitigation of the factors that aggravate this phenomenon.

## References

[1] Van de Vuurst P, Escobar LE (2023). Climate change and infectious disease: a review of evidence and research trends. Infect Dis Poverty.

[2] Booth M (2018). Climate change and the neglected tropical diseases. Adv Parasitol.

[3] Monaghan AJ, Sampson KM, Steinhoff DF, Ernst KC, Ebi KL, Jones B, Hayden MH (2018). The potential impacts of 21st century climatic and population changes on human exposure to the virus vector mosquito Aedes aegypti. Clim Change.

[4] Iwamura T, Guzman-Holst A, Murray KA (2020). Accelerating invasion potential of disease vector Aedes aegypti under climate change. Nat Commun.

[5] Agache I, Sampath V, Aguilera J (2022). Climate change and global health: a call to more research and more action. Allergy.

[6] Parums DV (2024). Editorial: climate change and the spread of vector-borne diseases, including dengue, malaria, Lyme disease, and West Nile virus infection. Med Sci Monit.

[7] Caminade C, McIntyre KM, Jones AE (2019). Impact of recent and future climate change on vector-borne diseases. Ann N Y Acad Sci.

[8] Tabassum S, Kalsoom T, Zaheer Z, Naeem A, Afifi A, Ohadi L (2023). Reflections on the surge in malaria cases after unprecedented flooding in Pakistan-a commentary. Health Sci Rep.

[9] Thomson MC, Stanberry LR (2022). Climate change and vectorborne diseases. N Engl J Med.

[10] Lee JS, Chung SY (2023). The threat of climate change on tick-borne infections: rising trend of infections and geographic distribution of climate risk factors associated with ticks. J Infect Dis.

[11] Gizaw Z, Salubi E, Pietroniro A, Schuster-Wallace CJ (2024). Impacts of climate change on water-related mosquito-borne diseases in temperate regions: a systematic review of literature and meta-analysis. Acta Trop.

[12] Chilakam N, Lakshminarayanan V, Keremutt S (2023). Economic burden of mosquito-borne diseases in low- and middle-income countries: protocol for a systematic review. JMIR Res Protoc.

[13] Kim CL, Agampodi S, Marks F, Kim JH, Excler JL (2023). Mitigating the effects of climate change on human health with vaccines and vaccinations. Front Public Health.

[14] Soto-Garita C, Murillo T, Chávez-Peraza I (2024). Epidemiological, virological and clinical characterization of a dengue/Zika outbreak in the Caribbean region of Costa Rica 2017-2018. Front Cell Infect Microbiol.

[15] Barboza LA, Chou-Chen SW, Vásquez P, García YE, Calvo JG, Hidalgo HG, Sanchez F (2023). Assessing dengue fever risk in Costa Rica by using climate variables and machine learning techniques. PLoS Negl Trop Dis.

[16] Aliaga-Samanez A, Romero D, Murray K, Segura M, Real R, Olivero J (2024). Potential climate change effects on the distribution of urban and sylvatic dengue and yellow fever vectors. Pathog Glob Health.

[17] Bonnin L, Tran A, Herbreteau V, Marcombe S, Boyer S, Mangeas M, Menkes C (2022). Predicting the effects of climate change on dengue vector densities in Southeast Asia through process-based modeling. Environ Health Perspect.

[18] Brustolin M, Pujhari S, Terradas G (2023). In vitro and in vivo coinfection and superinfection dynamics of Mayaro and Zika viruses in mosquito and vertebrate backgrounds. J Virol.

[19] Ohst C, Saschenbrecker S, Stiba K (2018). Reliable serological testing for the diagnosis of emerging infectious diseases. Adv Exp Med Biol.

[20] Kerkhof K, Falconi-Agapito F, Van Esbroeck M, Talledo M, Ariën KK (2020). Reliable serological diagnostic tests for arboviruses: feasible or utopia?. Trends Microbiol.

[21] Karan M, Paul S, Nath S (2024). One-step multiplex polymerase chain reaction assay for the detection of major disease-transmitting mosquito vectors in India. Am J Trop Med Hyg.

[22] Mojahed N, Mohammadkhani MA, Mohamadkhani A (2022). Climate crises and developing vector-borne diseases: a narrative review. Iran J Public Health.

[23] Chaves LF, Ramírez Rojas M, Prado M, Garcés JL, Salas Peraza D, Marín Rodríguez R (2020). Health policy impacts on malaria transmission in Costa Rica. Parasitology.

[24] Semenza JC, Rocklöv J, Ebi KL (2022). Climate change and cascading risks from infectious disease. Infect Dis Ther.

[25] Paz S (2024). Climate change: a driver of increasing vector-borne disease transmission in non-endemic areas. PLoS Med.

[26] Anikeeva O, Hansen A, Varghese B, Borg M, Zhang Y, Xiang J, Bi P (2024). The impact of increasing temperatures due to climate change on infectious diseases. BMJ.

[27] Baker RE, Mahmud AS, Miller IF (2022). Infectious disease in an era of global change. Nat Rev Microbiol.

[28] Akyea-Bobi NE, Akorli J, Opoku M (2023). Entomological risk assessment for transmission of arboviral diseases by Aedes mosquitoes in a domestic and forest site in Accra, Ghana. PLoS One.

[29] Buchwald AG, Hayden MH, Dadzie SK, Paull SH, Carlton EJ (2020). Aedes-borne disease outbreaks in West Africa: a call for enhanced surveillance. Acta Trop.

[30] Sargent K, Mollard J, Henley SF, Bollasina MA (2022). Predicting transmission suitability of mosquito-borne diseases under climate change to underpin decision making. Int J Environ Res Public Health.

[31] Barcellos C, Matos V, Lana RM, Lowe R (2024). Climate change, thermal anomalies, and the recent progression of dengue in Brazil. Sci Rep.

[32] Damtew YT, Tong M, Varghese BM (2023). Effects of high temperatures and heatwaves on dengue fever: a systematic review and meta-analysis. EBioMedicine.

[33] Smith MW, Willis T, Mroz E, James WH, Klaar MJ, Gosling SN, Thomas CJ (2024). Future malaria environmental suitability in Africa is sensitive to hydrology. Science.

[34] Nosrat C, Altamirano J, Anyamba A (2021). Impact of recent climate extremes on mosquito-borne disease transmission in Kenya. PLoS Negl Trop Dis.

[35] Franklinos L, Jones K, Readding D (2019). The effect of global change on mosquito-borne disease. Lancet.

[36] Wilcox BA, Echaubard P, de Garine-Wichatitsky M, Ramirez B (2019). Vector-borne disease and climate change adaptation in African dryland social-ecological systems. Infect Dis Poverty.

[37] Colón-González FJ, Gibb R, Khan K, Watts A, Lowe R, Brady OJ (2023). Projecting the future incidence and burden of dengue in Southeast Asia. Nat Commun.

[38] Gibb R, Colón-González FJ, Lan PT (2023). Interactions between climate change, urban infrastructure and mobility are driving dengue emergence in Vietnam. Nat Commun.

[39] Yu Q, Qu Y, Zhang L (2024). Spatial spillovers of violent conflict amplify the impacts of climate variability on malaria risk in sub-Saharan Africa. Proc Natl Acad Sci U S A.

[40] Alum EU, Tufail T, Agu PC, Akinloye DI, Obaroh IO (2024). Malaria pervasiveness in Sub-Saharan Africa: overcoming the scuffle. Medicine (Baltimore).

[41] Yan G, Lee MC, Zhou G (2022). Impact of environmental modifications on the ecology, epidemiology, and pathogenesis of Plasmodium falciparum and Plasmodium vivax malaria in East Africa. Am J Trop Med Hyg.

[42] Zhang Y, Wang M, Huang M, Zhao J (2024). Innovative strategies and challenges mosquito-borne disease control amidst climate change. Front Microbiol.

[43] Jones FK, Morrison AM, Santiago GA (2024). Introduction and spread of dengue virus 3, Florida, USA, May 2022-April 2023. Emerg Infect Dis.

[44] Burrows H, Talbot B, McKay R (2021). A multi-year assessment of blacklegged tick (Ixodes scapularis) population establishment and Lyme disease risk areas in Ottawa, Canada, 2017-2019. PLoS One.

[45] Ogden NH, Dumas A, Gachon P, Rafferty E (2024). Estimating the incidence and economic cost of Lyme disease cases in Canada in the 21st century with projected climate change. Environ Health Perspect.

[46] Ly H (2024). Dengue fever in the Americas. Virulence.

[47] Peng J, Zhang M, Wang G, Zhang D, Zheng X, Li Y (2024). Biased virus transmission following sequential coinfection of Aedes aegypti with dengue and Zika viruses. PLoS Negl Trop Dis.

[48] El-Sayed A, Kamel M (2020). Climatic changes and their role in emergence and re-emergence of diseases. Environ Sci Pollut Res Int.

[49] Mandage R, Kaur C, Pramanik A (2020). Association of dengue virus and Leptospira co-infections with malaria severity. Emerg Infect Dis.

[50] Noor S, Ismail M, Khadim F (2020). Potential drug-drug interactions associated with adverse clinical outcomes and abnormal laboratory findings in patients with malaria. Malar J.

[51] Gaspar-Castillo C, Rodríguez MH, Ortiz-Navarrete V, Alpuche-Aranda CM, Martinez-Barnetche J (2023). Structural and immunological basis of cross-reactivity between dengue and Zika infections: implications in serosurveillance in endemic regions. Front Microbiol.

[52] Medina FA, Vila F, Premkumar L (2021). Capacity of a multiplex IgM antibody capture ELISA to differentiate Zika and dengue virus infections in areas of concurrent endemic transmission. Am J Trop Med Hyg.

[53] Demers J, Bewick S, Agusto F, Caillouët KA, Fagan WF, Robertson SL (2020). Managing disease outbreaks: the importance of vector mobility and spatially heterogeneous control. PLoS Comput Biol.

[54] Horstick O, Runge-Ranzinger S (2018). Protection of the house against Chagas disease, dengue, leishmaniasis, and lymphatic filariasis: a systematic review. Lancet Infect Dis.

[55] Kumar G, Baharia R, Singh K (2024). Addressing challenges in vector control: a review of current strategies and the imperative for novel tools in India's combat against vector-borne diseases. BMJPH.

[56] Onyinyechi OM, Mohd Nazan AI, Ismail S (2023). Effectiveness of health education interventions to improve malaria knowledge and insecticide-treated nets usage among populations of sub-Saharan Africa: systematic review and meta-analysis. Front Public Health.

[57] Wilson AL, Courtenay O, Kelly-Hope LA, Scott TW, Takken W, Torr SJ, Lindsay SW (2020). The importance of vector control for the control and elimination of vector-borne diseases. PLoS Negl Trop Dis.

[58] Achee NL, Grieco JP, Vatandoost H (2019). Alternative strategies for mosquito-borne arbovirus control. PLoS Negl Trop Dis.

[59] Wang GH, Du J, Chu CY, Madhav M, Hughes GL, Champer J (2022). Symbionts and gene drive: two strategies to combat vector-borne disease. Trends Genet.

[60] Almeida L, Bellver-Arnau J, Privat Y, Rebelo C (2024). Vector-borne disease outbreak control via instant releases. J Math Biol.

[61] Moreno BJ, Aldridge RL, Britch SC (2021). Preparing irradiated and marked male Aedes aegypti mosquitoes for release in an operational sterile insect technique program. J Vis Exp.

[62] Chan EY, Sham TS, Shahzada TS (2020). Narrative review on health-EDRM primary prevention measures for vector-borne diseases. Int J Environ Res Public Health.

[63] Côrtes N, Lira A, Prates-Syed W (2023). Integrated control strategies for dengue, Zika, and chikungunya virus infections. Front Immunol.

